# The effectiveness of a percutaneous endoscopic approach in a patient with psoas and epidural abscess accompanied by pyogenic spondylitis: a case report

**DOI:** 10.1186/s13256-019-2193-6

**Published:** 2019-08-15

**Authors:** Keiichiro Iida, Koichi Yoshikane, Osamu Tono, Kiyoshi Tarukado, Katsumi Harimaya

**Affiliations:** 1 Department of Orthopaedic Surgery, Kyushu University Beppu Hospital, 4546 Tsurumibaru, Beppu, Oita 874-0838 Japan; 2 Department of Orthopaedic Surgery, Kitakyushu Municipal Medical Center, Fukuoka, Japan

**Keywords:** Percutaneous endoscopy, Psoas abscess, Epidural abscess, Pyogenic spondylitis, Drainage

## Abstract

**Background:**

Psoas or epidural abscesses are often accompanied by pyogenic spondylitis and require drainage. Posterolateral percutaneous endoscopic techniques are usually used for hernia discectomy, but this approach is also useful in some cases of psoas or lumbar ventral epidural abscess. We here report a case of psoas and epidural abscesses accompanied by pyogenic spondylitis that was successfully treated by percutaneous endoscopic drainage.

**Case presentation:**

Our patient was a 57-year-old Japanese woman who had been receiving chemotherapy for inflammatory breast cancer and who became unable to walk due to lower back and left leg pain. She was transported as an emergency to another hospital. Magnetic resonance imaging revealed psoas and epidural abscesses accompanied by pyogenic spondylitis, and methicillin-resistant *Staphylococcus aureus* was detected in a blood culture. Drainage of the psoas abscess was performed under echo guidance, but was not effective, and she was transferred to our institution. We performed percutaneous endoscopic drainage for the psoas and epidural abscesses. Immediate pain relief was achieved and the inflammatory reaction subsided after 8 weeks of antibiotic therapy with daptomycin.

**Conclusions:**

Percutaneous endoscopy allowed us to approach the psoas and epidural abscesses directly, enabling the immediate drainage of the abscesses with less burden on the patient.

## Background

Psoas abscesses are often accompanied by pyogenic spondylitis [1]. They can be treated with antibiotic therapy alone; however, drainage is recommended for cases involving large abscesses or when antibiotic therapy is ineffective [2]. Surgical drainage has been the traditional treatment; however, less invasive treatments, such as drainage under computed tomography (CT) or echo guidance, have become more common [3]. Open surgery is generally performed when percutaneous drainage is not effective. Posterolateral percutaneous endoscopic techniques are usually used for hernia discectomy [4]; however, this rare posterolateral approach is also useful in some cases of psoas or lumbar ventral epidural abscess. This technique enabled us to reach the abscess directly and to perform lavage and drainage less invasively in comparison to traditional open surgery. We here report a case of psoas and epidural abscesses in a patient with pyogenic spondylitis that were successfully treated by percutaneous endoscopic drainage.

## Case presentation

**Fig. 1:**
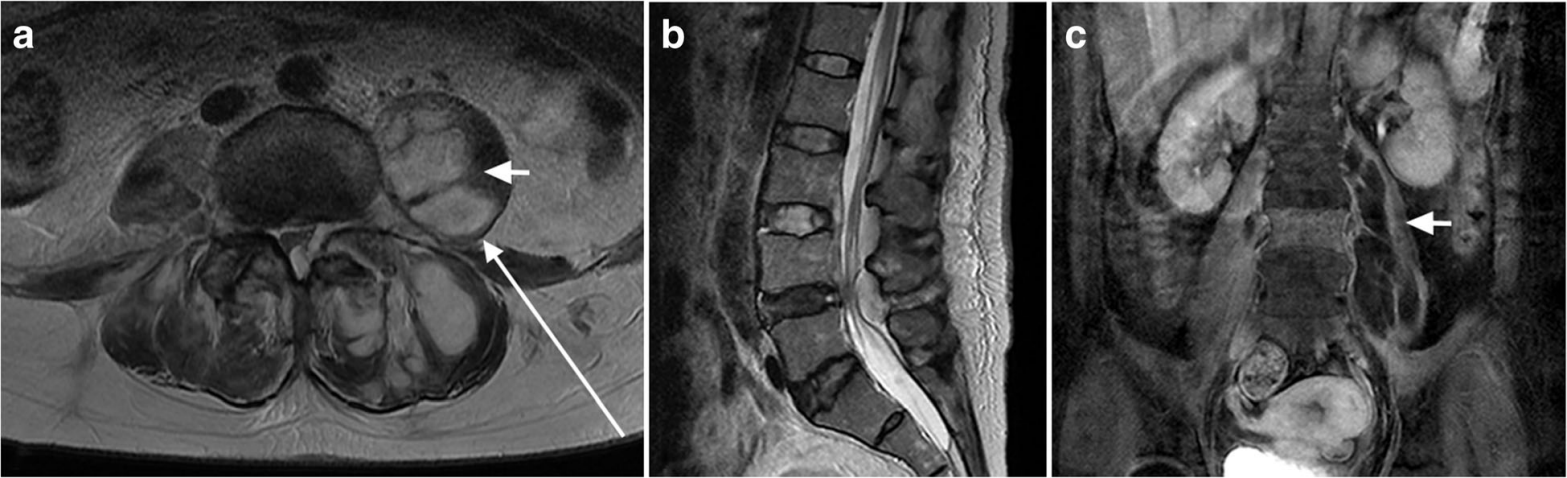
Magnetic resonance images of the lumbar vertebra before surgery. T2-weighted magnetic resonance imaging **a** L4–5 level, **b** sagittal view, and **c** coronal gadolinium-enhanced magnetic resonance imaging showing psoas, epidural, and paravertebral abscesses. The *short white arrow* shows the location of psoas abscess and the *long white arrow* shows the pathway of percutaneous endoscopy to the psoas abscess. The third vertebra is enhanced by gadolinium and diagnosed as pyogenic spondylitis (**c**)

**Fig. 2:**
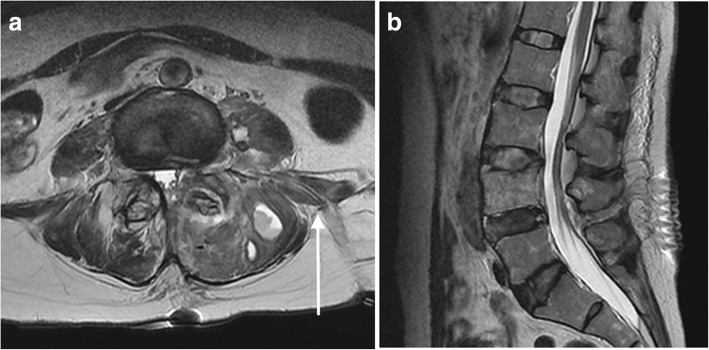
Magnetic resonance images of the lumbar vertebra at 1 week after surgery. **a** Axial and **b** sagittal T2-weighted magnetic resonance imaging showing the marked improvement of the psoas abscess. The *white arrow* shows the drainage tube from the psoas abscess

**Fig. 3:**
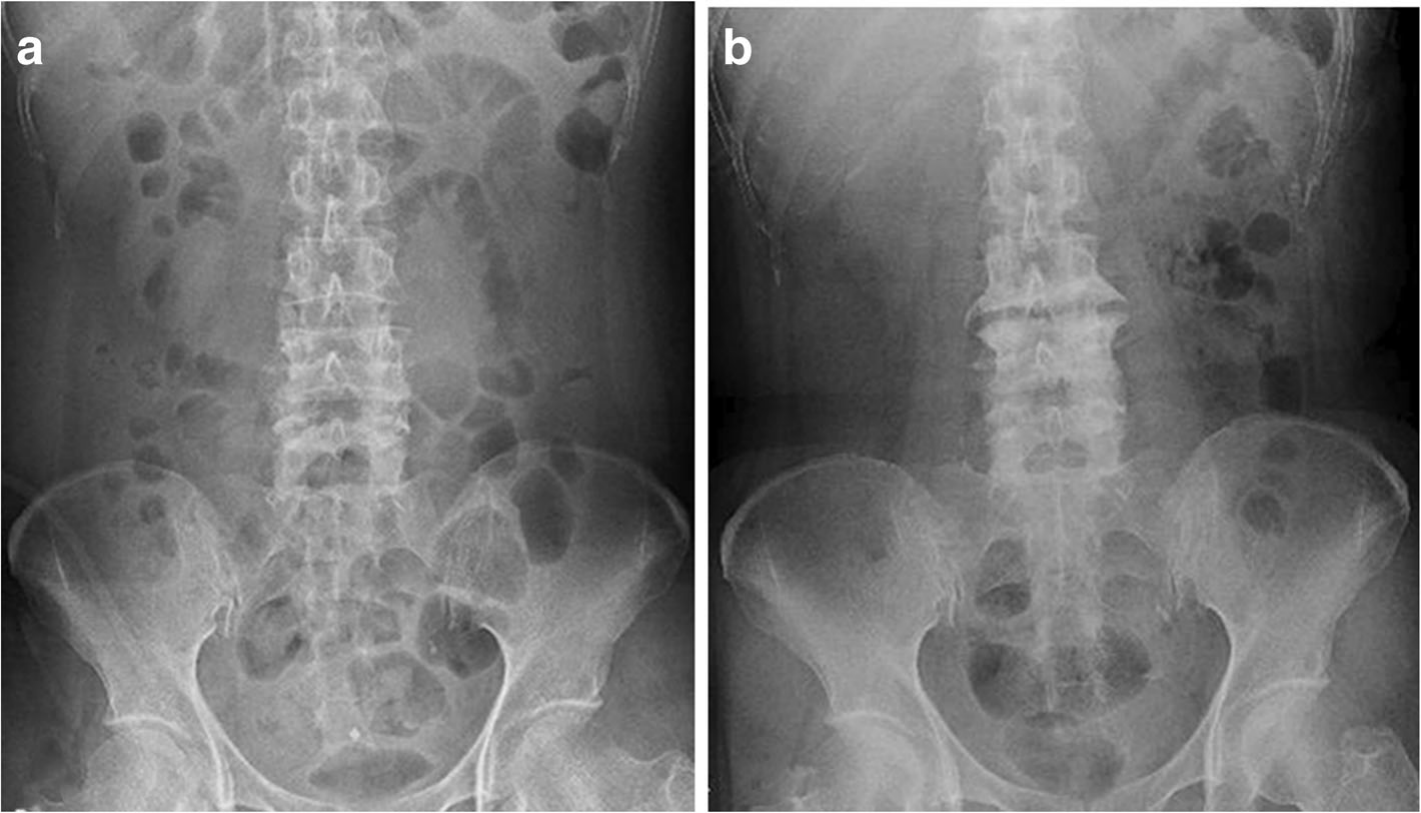
Plain radiographs show the degenerative change of the lumbar vertebra. Posteroanterior plain radiographs **a** before treatment and **b** at the final follow-up show the progression of degenerative change between the third and fourth lumbar vertebra

A 57-year-old Japanese woman who had been receiving chemotherapy for inflammatory breast cancer became unable to walk due to high fever and lower back and left leg pain. She was transported to another hospital as an emergency. Magnetic resonance imaging (MRI) revealed psoas and epidural abscesses accompanied by pyogenic spondylitis and methicillin-resistant *Staphylococcus aureus* (MRSA) was detected in a blood culture. A laboratory analysis revealed a white blood cell count of 17600/mm^3^, and a C-reactive protein level of 39.5 mg/L. The psoas abscess was drained under echo guidance. MRSA was also detected from the breast cancer ulcer and the aspirate of the psoas abscess. In spite of continuous drainage and antibiotic therapy with vancomycin, the abscess became larger and she was referred to our institution. On admission, she had a fever of 38.0 °C and her left leg was in the psoas position due to pain. Her manual muscle test (MMT) results were as follows: hip flexor, 5/3; knee extensor, 5/3; ankle extensor, 5/5; and ankle flexor, 5/5. A laboratory analysis revealed the following findings: white blood cell count, 10810/mm^3^ (neutrophils, 85%), C-reactive protein, 15.8 mg/L; total protein, 6.0 g/dl; and albumin, 1.6 g/dl. MRI demonstrated psoas and paravertebral abscesses, and epidural abscess with pyogenic spondylitis (Fig. 1). We performed percutaneous drainage for psoas and epidural abscess by endoscopy, and open drainage for paravertebral abscess under general anesthesia. At 1 week after drainage, the abscesses decreased to < 1 cm in size and the drain was removed (Fig. 2). The sensation around her knee weakened after surgery but the pain was immediately relieved. Antibiotic therapy with daptomycin was continued for 8 weeks. She regained the ability to walk and the inflammatory reaction subsided. The hypesthesia around her knee recovered. No recurrence of pyogenic spondylitis or psoas abscess was observed. At the final follow-up, the pyogenic spondylitis was healed with degenerative change between the third and fourth lumbar vertebra (Fig. 3). She died of breast cancer 1 year after surgery.

### The endoscopic surgical procedure

**Fig. 4:**
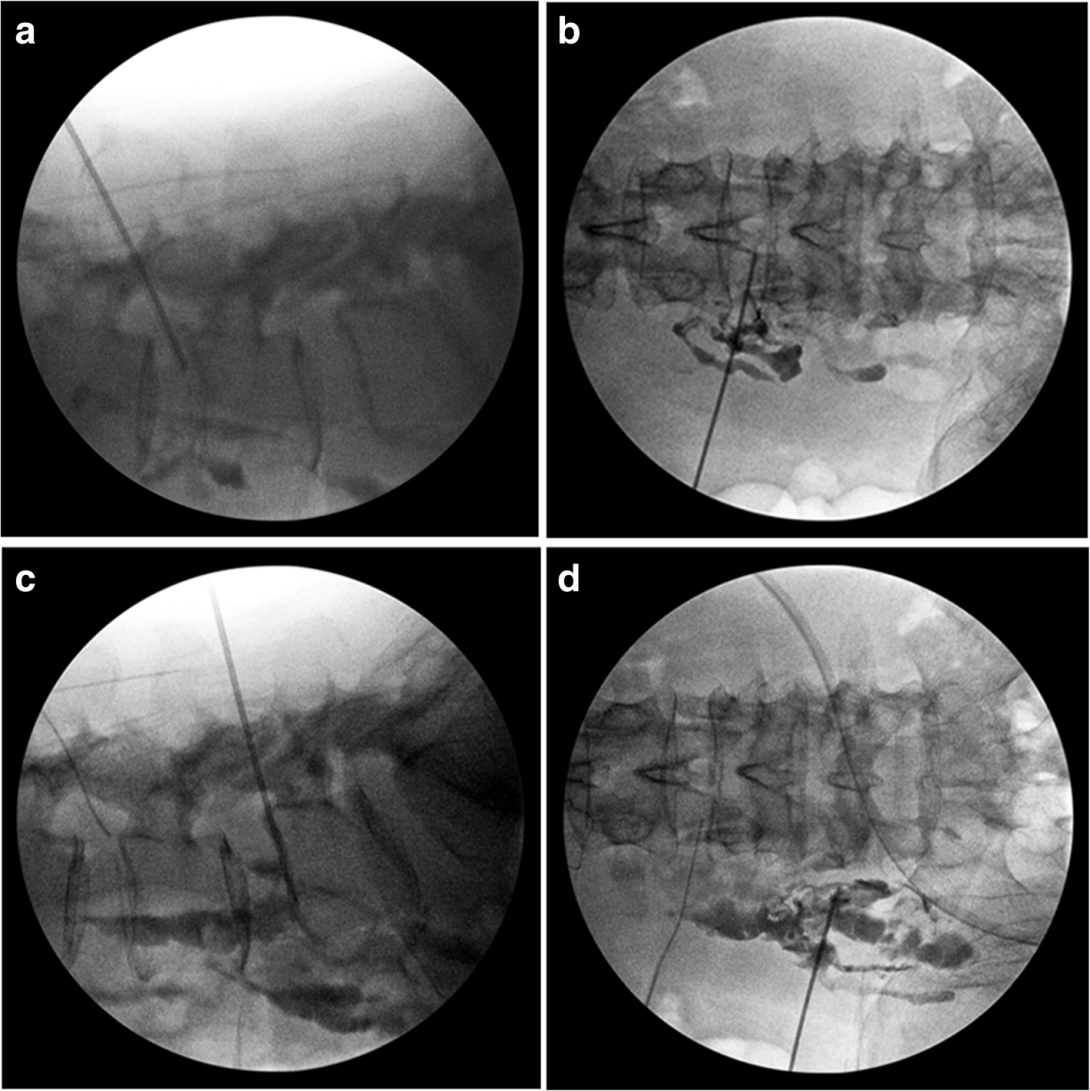
Fluoroscopy images during surgery. **a**, **b** The passage between the lumbar disc and the psoas abscess was detected by the flow of Omnipaque (iohexol). **c**, **d** The position of needle in the psoas muscle was confirmed by the flow of Omnipaque (iohexol)

**Fig. 5:**
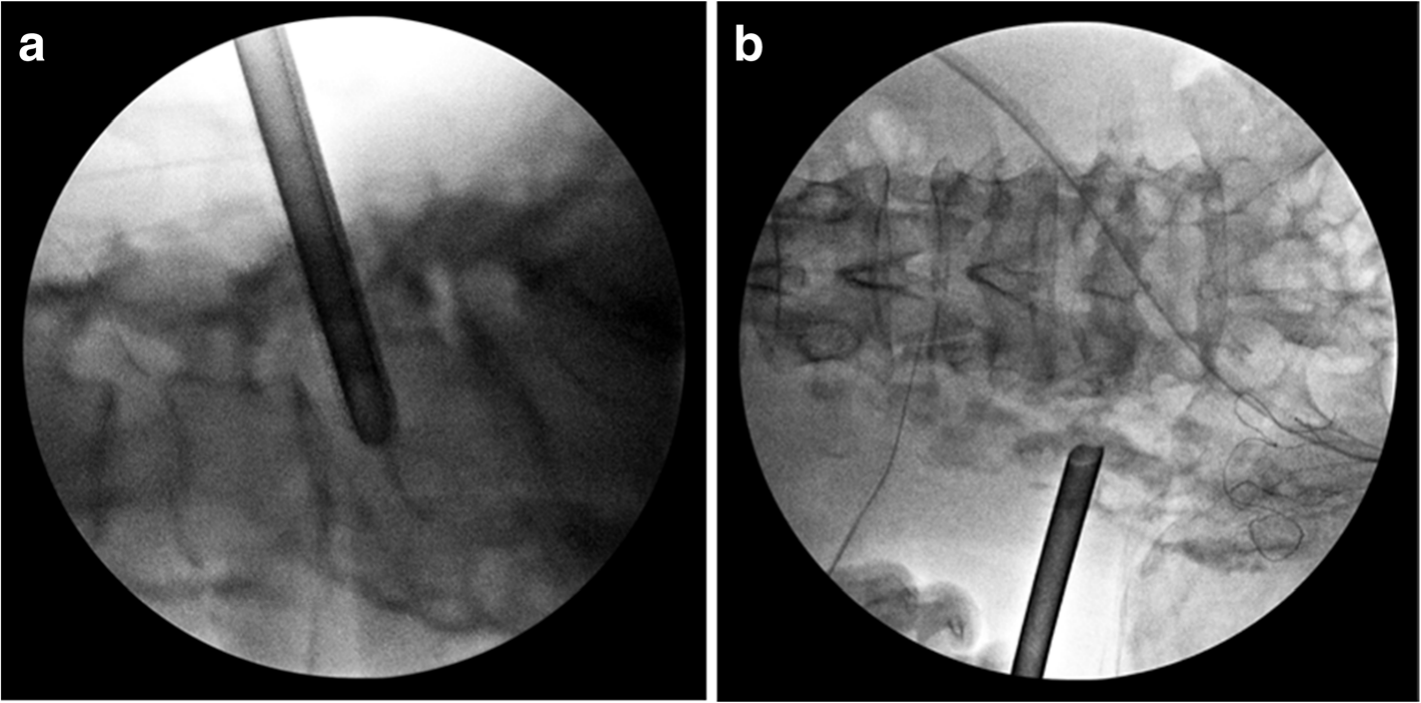
Fluoroscopy images during surgery. **a**, **b** The position of the cannula was confirmed to prevent it from moving in a ventral direction during surgery

Our patient was placed prone on a Jackson Table for a fluoroscopy. All procedures were performed under total intravenously administered anesthesia. A spinal needle was inserted into the target disk from a point 10 cm from the midline. Indigo carmine and Omnipaque (iohexol) were injected into the disc. The passage between the lumbar disc and the psoas abscess was detected by the flow of Omnipaque (iohexol) on fluoroscopy (Fig. 4). A dilator and elevated working sleeve were guided over the needle and into the disc space. The dilator was removed and a cutting tool was inserted. A piece of disc was removed and the cannula was pulled to find the epidural space. The epidural space above the posterior longitudinal ligament was felt carefully by spatula and the pus was evacuated and irrigated. The water pressure was set to 30 mmHg. To drain the psoas abscess, the same procedure was performed. A spinal needle was inserted into the psoas muscle from a point 10 cm lateral from the midline. Under fluoroscopy, the spinal needle was inserted to the middle depth of the fourth lumbar vertebra at the transverse process level of the fourth lumbar vertebra. The placement was confirmed to be safe by preoperative MRI. Indigo carmine and Omnipaque (iohexol) were injected to confirm that the needle was located in the psoas muscle (Fig. 4). An elevated-type cannula was impacted and inserted into the psoas muscle. A large amount of pus flowed from the cannula. The psoas muscle was felt carefully by spatula and the position of the cannula was confirmed by fluoroscopy to ensure that it did not move ventrally (Fig. 5). After irrigation, suction drains were placed in the spinal disc space and the psoas muscle.

## Discussion

Psoas abscesses are classified as primary or secondary; pyogenic spondylitis is one of the etiologies of secondary psoas abscess. When a psoas abscess is small, antibiotic therapy alone can be selected; however, when the abscess becomes large, drainage is recommended. Percutaneous drainage under CT or echo guidance is generally used for the drainage of psoas abscesses; however, drainage fails in a considerable number of cases, especially in cases involving multiloculated abscess cavities or with thick tenacious pus [5]. In such cases, an extraperitoneal approach has traditionally been performed. We used a posterolateral endoscopic approach to reduce the invasiveness of surgery as this patient was in a septic condition with severe undernutrition.

There are some reports on the performance of posterolateral percutaneous endoscopic surgery for the treatment of pyogenic spondylitis [6, 7]. The case series demonstrated that this approach provided acute pain relief and the early subsidence of spinal infection. It is difficult to judge the need of the surgical intervention because the cases may be treatable by percutaneous drainage under CT or echo guidance. Our case demonstrated that this percutaneous drainage technique can be adopted in the failure cases of percutaneous drainage, and showed that we could approach a psoas abscess directly by percutaneous endoscopy. The injection of Omnipaque (iohexol) into the lumbar disc before surgery proved the passage of pus between the lumbar disc and the psoas abscess; however, we decided to drain the psoas abscess directly as the abscess size was so large that setting the suction tube in the lumbar disc would probably have been insufficient for treating the psoas abscess. The approach to the psoas muscle is the same approach that is used in lumbar plexus blockade [8]. We only used fluoroscopy because the preoperative images showed that no organs were in the way of the approach; however, echo guidance allows for the safer insertion of a needle into the psoas. Preoperative CT or MRI images can reveal when this approach would be difficult, and in which traditional open surgery would be needed. The advantage of percutaneous drainage in comparison to drainage under CT or echo guidance is that the abscess can be irrigated directly with a large amount of water and a thicker tube can be placed. This procedure is considered to contribute to immediate pain relief and an acute decrease in the size of the abscess. MRI at 1 week after surgery showed that the abscess was almost completely diminished.

Some precautions should be taken in this approach. Our patient complained of mild hypesthesia around her left knee after surgery. This may have been an after effect of the epidural and psoas abscesses, or it may have occurred due to exiting nerve root damage that was caused by the transforaminal approach for epidural abscess drainage [9], or because of the lumbar plexus damage that occurred during the direct approach to the psoas abscess [10]. It was difficult to identify the cause of hypesthesia as many factors were present. Our technique of treating the psoas abscess simply involved perforating the psoas muscle and feeling the inside with a spatula; thus, the risk of damaging the lumbar plexus is considered to be low. However, it is important to keep in mind the risk of damage to the lumbar plexus when we directly approach the psoas muscle. In addition, we need to monitor the position of the cannula by fluoroscopy in order to prevent it from moving ventrally and to avoid injuring the peritoneum or urinary tract during surgery. Even though there were some technical points that needed to be remembered, the operative invasiveness was low in comparison to conventional open surgery and it was immediately effective. Moreover, while ventral epidural abscesses are difficult to drain by traditional open surgery, this unusual approach enabled us to approach the site easily. We performed this surgery under general anesthesia because three skin incisions were needed; however, the procedure is generally performed under local anesthesia to avoid exiting nerve injury. Patients with pyogenic spondylitis who have several abscesses are generally in an immunosuppressive state and the condition has the potential to be life threatening. This procedure could be a treatment option even for patients in a poor general condition and for whom general anesthesia would be considered to be associated with a high degree of risk.

Six weeks of antibiotic therapy is recommended for the treatment of pyogenic spondylitis, and more long-term antibiotic treatment is recommended for MRSA infection [11]. Daptomycin seemed more effective than vancomycin for MRSA [12, 13]. We administered antibiotic therapy for 8 weeks after surgery, which led to a reduction in the inflammatory reaction. No marked vertebral destruction was observed, and our patient was a good candidate for endoscopic treatment. As mentioned in previous reports, some surgical instruments or anterior column reconstruction may be necessary when pyogenic spondylitis is accompanied by severe vertebral destruction [6].

## Conclusions

Percutaneous endoscopy allowed us to approach the psoas and epidural abscesses directly, enabling the immediate drainage of the abscesses with less burden on the patient. It was suggested that a percutaneous endoscopic approach is one of the effective treatments for psoas and epidural abscesses.

## Data Availability

All data generated or analyzed during this study are included in this published article.
